# Topical Treatment of Parenchymatous Keratitis and Corneal Opacities with Mercurial Ointment

**Published:** 1892-09

**Authors:** 


TOPICAL TREATMENT OF PARENCHYMATOUS KERATITIS AND CORNEAL OPACITIES WITH MERCURIAL OINTMENT.—J. Mitvalsky, M. D. (*Merck's Bulletin*, May, 1892,) writes of the treatment of parenchymatous keratitis and corneal opacities with a diluted mercurial ointment. The formula he uses is:

| | |
|---|---|
| Mercurial ointment (33 per cent.), | 1 part. |
| Vaseline, | 2 parts. |
| Lanolin, | 1 part. |

He finds the greatest amount of good result from the use of the ointment in parenchymatous keratitis in the very first stages of infiltration. The application of the ointment causes a prompt absorption of the products of infiltration in most cases without a typical vascular stage developing. If there is much pericorneal injection, brow-ache, or photophobia, the ointment is contra-indicated. Good results are obtained only when the inflammation is accompanied by irritation or very mild symptoms of ciliary irritation. When the inflammatory process is declining, the ointment is valuable. In clearing up old corneal opacities, he regards the ointment as superior to any agent we possess.

